# Anthropometry, body fat composition and reproductive factors and risk of oesophageal and gastric cancer by subtype and subsite in the UK Biobank cohort

**DOI:** 10.1371/journal.pone.0240413

**Published:** 2020-10-20

**Authors:** Harinakshi Sanikini, David C. Muller, Marc Chadeau-Hyam, Neil Murphy, Marc J. Gunter, Amanda J. Cross

**Affiliations:** 1 Department of Epidemiology and Biostatistics, School of Public Health, Imperial College London, London, United Kingdom; 2 Section of Nutrition and Metabolism, International Agency for Research on Cancer, Lyon, France; University of Zurich, SWITZERLAND

## Abstract

**Background:**

Obesity has been positively associated with upper gastrointestinal cancers, but prospective data by subtype/subsite are limited. Obesity influences hormonal factors, which may play a role in these cancers. We examined anthropometry, body fat and reproductive factors in relation to oesophageal and gastric cancer by subtype/subsite in the UK Biobank cohort.

**Methods:**

Among 458,713 UK Biobank participants, 339 oesophageal adenocarcinomas, 124 oesophageal squamous cell carcinomas, 137 gastric cardia and 92 gastric non-cardia cancers were diagnosed during a mean of 6.5 years follow-up. Cox models estimated multivariable hazard ratios (HRs) and 95% confidence intervals (CIs).

**Results:**

Body mass index (BMI), hip circumference, waist circumference, waist-to-hip ratio, waist-to-height ratio, total body fat and trunk fat were positively associated with oesophageal adenocarcinoma (highest vs lowest category: HR = 2.33, 95%-CI:1.65–3.28; HR = 1.56, 95%-CI:1.15–2.13; HR = 2.30, 95%-CI:1.47–3.57; HR = 1.71, 95%-CI:1.01–2.90; HR = 2.87, 95%-CI:1.88–4.38; HR = 1.96, 95%-CI:1.30–2.96; HR = 2.34, 95%-CI:1.70–3.22, respectively). Although there were no statistically significant associations in combined sex analyses, BMI (HR = 1.83, 95%-CI:1.00–3.37), waist circumference (HR = 2.21, 95%-CI:1.27–3.84) and waist-to-hip ratio (HR = 1.92, 95%-CI:1.11–3.29) were associated with gastric cardia cancer in men; however, mutual adjustment attenuated the associations for BMI and waist-to-hip ratio. For oesophageal squamous cell carcinoma, statistically significant inverse associations were observed among women for BMI, hip circumference, waist circumference, waist-to-height ratio, total body fat and trunk fat, although they were based on small numbers. In addition, older age at first (HR = 0.44, 95%-CI:0.22–0.88) and last live birth (HR = 0.44, 95%-CI:0.22–0.87) were inversely associated with oesophageal squamous cell carcinoma and having a stillbirth/miscarriage/termination was positively associated (HR = 1.84, 95%-CI:1.10–3.07).

**Conclusions:**

Obesity and abdominal obesity specifically may be a risk factor for oesophageal adenocarcinoma and gastric cardia cancer in men. Some reproductive factors may be associated with oesophageal squamous cell carcinoma in women.

## Introduction

Globally, oesophageal and gastric cancers are the seventh and fifth most common cancers, with an estimated 572,000 and 1,000,000 cases in 2018, respectively [1]. Both cancer types are more common in men than in women [1]. The aetiology of oesophageal cancer differs by the two main histological subtypes of adenocarcinoma and squamous cell carcinoma. Gastro-oesophageal reflux disease, smoking and obesity are recognized risk factors for oesophageal adenocarcinoma, whereas smoking and alcohol are well-known risk factors for oesophageal squamous cell carcinoma [2]. Similarly, gastric cancers appear to have distinct aetiologies by anatomical subsite, which are classified as gastric cardia and gastric non-cardia. Smoking and obesity are known risk factors for gastric cardia cancer, while *Helicobacter Pylori* infection and smoking are risk factors for gastric non-cardia cancer [3].

A number of epidemiological studies have reported a positive association between body mass index (BMI) and risk of oesophageal adenocarcinoma and gastric cardia cancer [4–6]. In contrast, an inverse association between BMI and oesophageal squamous cell carcinoma has been reported in some studies [2, 7–9]. However, prospective cohort studies examining abdominal obesity in relation to oesophageal and gastric cancer by subtype and subsite are limited, with conflicting findings [8, 10–12]. Furthermore, to our knowledge, only one cohort study has investigated body fat composition measurements that distinguish between adipose and non-adipose mass (estimated using bioelectrical impedance) in relation to oesophageal and gastric cancer risk [10].

There are several plausible biological mechanisms linking obesity to upper gastrointestinal cancers. Obesity can lead to metabolic disturbances, such as higher levels of pro inflammatory cytokines (e.g. tumour necrosis factor-α and interleukin-6), adipokines (e.g. glucose, insulin, and leptin), and endogenous sex steroids, which may increase cancer risk [13].

There is substantial evidence for sex differences in body fat distribution [14]. Men have a tendency to accumulate more visceral fat, while women store more fat in the subcutaneous depot [14]. Sex hormones play a role in body fat distribution [15]. Oestrogen promotes the accumulation of fat in the subcutaneous depot rather than to the visceral fat depot and the decrease in oestrogen levels in menopausal women is associated with an increase in visceral fat [15]. In addition to the regulation of body fat distribution, sex hormones may also explain the predominance of oesophageal and gastric cancers in men compared to women. It has been suggested that sex hormones, particularly oestrogens, may protect against the development of oesophageal and gastric cancer [16, 17]. Several epidemiological studies have investigated the role of hormonal and reproductive factors in the development of oesophageal and gastric cancer risk [18–22]; however, few prospective studies have examined the association between reproductive factors and risk of oesophageal and gastric cancer by subtype and subsite and the published findings are inconsistent [23–27].

The current study investigated the associations between anthropometric and body fat composition measurements, as well as reproductive factors with the risk of oesophageal and gastric cancer by subtype and subsite in the UK Biobank cohort.

## Materials and methods

### Study population

The UK Biobank study is a large prospective cohort consisting of 502,524 men and women, aged 40–69 years, recruited between 2006 and 2010 from 22 study centres across the UK; more details of the rationale and study design have been published previously [28, 29]. Participants were identified from National Health Service patient registers. At recruitment, participants were asked to complete a touchscreen self-administered questionnaire that included questions on socio-demographic factors, health and medical history, lifestyle exposures, early-life exposures, and medication use. For this study, we excluded participants with prevalent cancer at recruitment (n = 43,811); leaving n = 458,713 for analysis.

Ethical approval of the UK Biobank cohort was obtained from the North West Multi-centre Research Ethics Committee, the National Information Governance Board for Health and Social Care in England and Wales, and the Community Health Index Advisory Group in Scotland. All participants signed an informed consent form.

### Anthropometric and body fat composition measurements

At baseline, trained staff measured standing height using the Seca 202 device and body weight using the Tanita BC-418MA [28]. The Wessex non-stretchable sprung tape measure was used to measure waist and hip circumference. BMI was calculated as weight in kilograms divided by height in meters squared, waist-to-hip ratio was computed as waist circumference (cm) divided by hip circumference (cm) and waist-to-height ratio was computed as waist circumference (cm) divided by height (cm). The Tanita BC-418MA body composition analyser was used to assess bioelectrical impedance measures including total body fat percentage and trunk fat percentage. Measurements were performed on participants in light clothing after removal of shoes and heavier outer clothes. Participants were asked to stand briefly in bare feet on the analysers footpads and hold the handles where measurements of bio-impedance were taken.

### Reproductive factors

Information on reproductive factors was collected at baseline. The following reproductive characteristics were assessed: age at menarche, ever had stillbirth/miscarriage/termination, number of live births, age at first live birth, age at last live birth, age at menopause, ever taken oral contraceptive pills, age started oral contraceptive pills, age last used oral contraceptive pills, and ever used hormone replacement therapy. Due to a small number of cases in some of the categories, we did not have enough power to present data for age at menopause and for oral contraceptive pill use.

### Ascertainment of cancer cases

Participants were followed via record linkage to UK National Health Service Registers. Cancer cases were identified through linkage to national cancer registries annually. Complete follow-up was available until 2016. Only 0.26% of participants were lost to follow-up. First primary incident oesophageal and gastric cancers were coded according to the 10^th^ revision of the International Classification of Diseases (ICD-10). Oesophageal cancer included ICD for Oncology (ICD-O) topography codes C15.0-C15.9; oesophageal adenocarcinoma was classified as ICD-O morphological codes: 8140, 8141, 8190–8231, 8260–8263, 8310, 8430, 8480–8490, 8560, 8570–8572 and oesophageal squamous cell carcinoma was classified as ICD-O morphological codes: 8050–8076. Gastric cancer was restricted to adenocarcinomas and included topography ICD-O codes: C16; gastric cardia was categorized as C16.0 and gastric non-cardia included C16.1–16.6.

### Statistical analysis

Hazard ratios (HRs) and 95% confidence intervals (CIs) were computed based on Cox proportional hazard models, with age as the primary time variable. The study entry time was defined as age at recruitment and exit time as age at diagnosis (cases) or censoring (date of death, diagnosis of other cancers or last date at which follow-up was considered complete). We tested the proportional hazards assumption using Schoenfeld residuals. Models were stratified by age at recruitment in five year categories, Townsend Deprivation Index [30] (quintiles), and recruitment assessment centre. The Townsend Deprivation Index score is an indicator of socioeconomic status and it was derived from national census data on car ownership, household overcrowding, owner occupation, and unemployment data that had been aggregated for postcodes of residence [31].

#### Anthropometric and body fat composition variables

Analyses were carried out for both sexes combined and separately for men and women. Given that men and women have different body fat distributions, sex-specific tertiles were used for anthropometric variables (height, weight, waist circumference, hip circumference, waist-to-hip ratio and waist-to-height ratio) and body fat composition variables (body fat percentage and trunk fat percentage). Tertiles were selected based on the distribution in the total cohort. For anthropometric and body fat composition variables, those with missing values were assigned to a “missing” category. For instance, we classified BMI into five categories of which four were defined according to World Health Organization categories and one as a missing category [underweight (BMI <18.5 kg/m^2^), normal weight (18.5 ≤ BMI <25 kg/m^2^), overweight (25 ≤ BMI <30 kg/m^2^), obese (≥30 kg/m^2^) and missing].

Baseline characteristics of study participants were assessed by X^2^ test for categorical variables and Kruskal-Wallis test for continuous variables. Covariates were selected based on existing evidence for risk factors of upper gastrointestinal cancers or if they resulted in a change of 10% or more in the risk estimates. Models were adjusted for smoking status (never, former and current), and education (none; CSEs/O levels/GCSEs [Certificate of Secondary Education/General Certificate of Secondary Education or equivalent]; vocational qualifications [National Vocational Qualification/Higher National Diploma/Higher National Certificate, A levels/Advanced Subsidiary levels or equivalent]; other qualifications; college/university degree; unknown). Models for oesophageal squamous cell carcinoma were also adjusted for alcohol intake frequency (never; special occasions only; 1–3 times/month, 1–2 times/week; 3–4 times a week; daily or almost daily; unknown). Additional models were investigated in which BMI and waist-to-hip ratio (categorical) were mutually adjusted, to assess whether abdominal obesity specifically was related to upper gastrointestinal cancers independently of overall obesity. We also examined separate models where waist circumference and hip circumference (categorical) were mutually adjusted for each other. We explored interactions for anthropometric variables with sex and smoking status by including an interaction term along with the main effect term in the adjusted model. The statistical significance of the interaction term was assessed using likelihood ratio tests.

#### Reproductive factors

These variables were classified into categories as follows: age at menarche (<13, ≥13 years), ever had stillbirth/miscarriage/termination (yes/no), number of live births (<2, ≥2 births), age at first live birth (<25, ≥25 years), age at last live birth (<30, ≥30 years), and ever used hormone replacement therapy (yes/no). Mean and standard deviations or frequencies were computed for baseline characteristics in women stratified by hormonal replacement therapy. Models for oesophageal adenocarcinoma, gastric cardia and non-cardia cancer were adjusted for smoking status, education and BMI; models for oesophageal squamous cell carcinoma were additionally adjusted for alcohol intake frequency. An indicator category was created for missing data on covariates.

Tests for linear trend were performed across categories of anthropometric, body fat composition and reproductive variables by excluding the missing values and allocating the median value to each category as a continuous term in the Cox regression models. Sensitivity analyses included a one-year lag analysis and a model where we applied tighter control for smoking by adding a pack-years variable to the model. P-values <0.05 were considered statistically significant, and all analyses were performed using SAS 9.4 software (SAS Institute, Cary, NC).

## Results

After a mean follow-up of 6.5 years, 339 oesophageal adenocarcinoma (291 men and 48 women), 124 oesophageal squamous cell carcinoma (54 men and 70 women), 137 gastric cardia (113 men and 24 women) and 92 gastric non-cardia (57 men and 35 women) cases were diagnosed among the 458,713 participants (214,293 men and 244,420 women).

### Anthropometric measurements

According to BMI categories in the cohort, 0.5% participants were underweight, 32.1% were normal weight, 42.3% were overweight and 24.3% were obese. Compared with normal weight subjects, obese men and women were slightly older, had a higher waist circumference, hip circumference, body fat, and trunk fat; furthermore, they were less educated, less likely to be smokers and less likely to drink alcohol (S1 Table).

In the combined analysis of men and women, several anthropometric measurements were positively associated with oesophageal adenocarcinoma (Table 1), including weight (HR 1.71, 95% CI: 1.17–2.50 for >84 vs <70 kg), BMI (HR 2.33, 95% CI: 1.65–3.28 for obese vs normal weight), hip circumference (HR 1.56, 95% CI: 1.15–2.13 for >106 vs <99 cm), waist circumference (HR 2.30, 95% CI: 1.47–3.57 for >96 vs <84 cm), waist-to-hip ratio (HR 1.71, 95% CI:1.01–2.90 for >0.92 vs <0.83) and waist-to-height ratio (HR 2.87, 95% CI: 1.88–4.38 for >0.56 vs <0.50). Furthermore, total percent body fat and trunk fat percentage were positively associated with oesophageal adenocarcinoma (HR 1.96, 95% CI: 1.30–2.96 for body fat >35.3 vs <26.9% and HR 2.34, 95% CI: 1.70–3.22 for trunk fat >34.5 vs <27.7%) (Table 1). Conversely, statistically significant inverse associations were observed for oesophageal squamous cell carcinoma with height, weight, BMI, hip circumference, waist circumference, waist-to-height ratio, and body fat and trunk fat percentage (Table 1). No statistically significant associations were observed with gastric cardia cancer, while for gastric non-cardia cancer inverse associations were observed with hip circumference (HR 0.53, 95% CI: 0.30–0.93 for >106 vs <99 cm) and trunk fat percentage (HR 0.50, 95% CI: 0.27–0.94 for >34.5 vs <27.7%) (Table 1). When we examined a model containing all of the anthropometric measures, no additional statistically significant associations were observed (Table 1).

**Table 1:** Adjusted hazard ratios (HR) and 95% confidence intervals (CI) for oesophageal and gastric cancer by subtype and subsite in men (n = 214,293) and women (n = 244,420) according to anthropometric factors in the UK Biobank cohort.

** **	** **		**Oesophageal cancer**			**Gastric cancer**		** **
	Adenocarcinoma		Squamous cell carcinoma		Cardia		Non-cardia	
	Cases/Non-cases	Adjusted HR^a^ (95% CI)	Cases/Non-cases	Adjusted HR^b^ (95% CI)	Case/Non-cases	Adjusted HR^a^ (95% CI)	Cases/Non-cases	Adjusted HR^a^ (95% CI)
**Height (cm)**								
**<164**	39/146,564	Reference	50/146,553	Reference	15/146,588	Reference	28/146,575	Reference
**164–173**	151/166,765	1.28 (0.84–1.96)	48/166,868	0.75 (0.46–1.23)	57/166,859	2.22 (1.05–4.71)	27/166,889	0.69 (0.31–1.18)
**>173**	144/142,002	0.99 (0.63–1.57)	21/142,125	0.40 (0.20–0.82)	62/142,084	2.24 (0.99–5.05)	36/142,110	0.93 (0.43–2.03)
**Missing**	5/3043	1.10 (0.24–5.10)	5/3043	1.90 (0.45–8.09)	3/3045	-	1/3047	-
*P*_*trend*_		0.09		0.04		0.11		0.15
**Weight (kg)**								
**<70**	44/149,838	Reference	62/149,820	Reference	20/149,862	Reference	22/149,860	Reference
**70–84**	107/155,714	1.27 (0.86–1.87)	34/155,787	0.50 (0.32–0.80)	43/155,778	1.16 (0.64–2.11)	32/155,789	1.00 (0.56–1.80)
**>84**	182/149,588	1.71 (1.17–2.50)	23/149,747	0.34 (0.19–0.59)	70/149,700	1.61 (0.89–2.91)	37/149,733	1.13 (0.62–2.09)
**Missing**	6/3234	1.96 (0.55–6.29)	5/3235	1.48 (0.36–6.07)	4/3236	2.15 (0.28–16.58)	1/3239	-
*P*_*trend*_		0.006		0.0003		0.14		0.87
BMI (kg/m^2^) ^c^								
Underweight^d^	1/2326	-	4/2323	-	1/2326	-	2/2325	-
**Normal weight**	51/147,311	Reference	45/147,317	Reference	30/147,332	Reference	28/147,334	Reference
**Overweight**	153/193,676	1.54 (1.10–2.15)	54/193,775	0.90 (0.59–1.36)	60/193,769	1.13 (0.71–1.82)	38/193,791	0.74 (0.45–1.23)
**Obese**	128/111,520	2.33 (1.65–3.28)	16/111,632	0.42 (0.23–0.79)	42/111,606	1.32 (0.79–2.21)	23/111,625	0.74 (0.42–1.32)
**Missing**	6/3541	2.06 (0.59–7.11)	5/3542	1.87 (0.46–7.68)	4/3543	1.77 (0.24–13.23)	1/3546	-
*P*_*trend*_		<0.0001		0.02		0.55		0.45
**Hip circumference (cm)**								
**<99**	70/138,315	Reference	57/138,328	Reference	31/138,354	Reference	34/138,351	Reference
**99–106**	138/179,689	1.17 (0.86–1.57)	44/179,783	0.60 (0.40–0.91)	54/179,773	1.16 (0.73–1.85)	35/179,792	0.68 (0.42–1.11)
**>106**	126/137,650	1.56 (1.15–2.13)	20/137,756	0.35 (0.20–0.60)	49/137,727	1.34 (0.82–2.19)	22/137,754	0.53 (0.30–0.93)
**Missing**	5/2720	1.48 (0.32–6.88)	3/2722	0.21 (0.01–6.35)	3/2722	-	1/2724	-
*P*_*trend*_		0.008		0.0003		0.49		0.07
**Waist circumference (cm)**								
**<84**	28/146,923	Reference	49/146,902	Reference	19/146,932	Reference	21/146,930	Reference
**84–96**	104/166,015	1.41 (0.90–2.22)	42/166,077	0.81 (0.51–1.29)	39/166,080	0.82 (0.44–1.51)	33/166,086	0.95 (0.52–1.73)
**>96**	202/142,768	2.30 (1.47–3.57)	30/142,940	0.55 (0.32–0.95)	76/142,894	1.28 (0.70–2.32)	37/142,933	0.96 (0.51–1.81)
**Missing**	5/2668	2.16 (0.45–10.43)	3/2670	0.30 (0.01–7.39)	3/2670	-	1/2672	-
*P*_*trend*_		<0.0001		0.09		0.09		0.98
**Waist to hip ratio**								
**<0.83**	25/146,314	Reference	39/146,300	Reference	18/146,321	Reference	19/146,320	Reference
**0.83–0.92**	99/174,433	1.24 (0.75–2.05)	45/174,487	0.99 (0.61–1.62)	35/174,497	0.59 (0.29–1.19)	30/174,502	0.95 (0.49–1.84)
**>0.92**	210/134,875	1.71 (1.01–2.90)	37/135,048	1.03 (0.55–1.91)	81/135,004	1.01 (0.49–2.09)	42/135,043	1.10 (0.52–2.34)
**Missing**	5/2752	1.68 (0.99–2.85)	3/2754	0.40 (0.02–9.15)	3/2754	-	1/2756	-
*P*_*trend*_		0.02		0.99		0.04		0.87
**Waist to height ratio**								
**<0.50**	28/140,899	Reference	45/140,882	Reference	20/140,907	Reference	21/140,906	Reference
**0.50–0.56**	113/169,080	1.70 (1.10–2.63)	44/169,149	0.76 (0.49–1.19)	50/169,143	1.12 (0.64–1.96)	38/169,155	1.01 (0.57–1.77)
**>0.56**	193/145,244	2.87 (1.88–4.38)	30/145,407	0.51 (0.31–0.86)	64/145,373	1.27 (0.73–2.22)	32/145,405	0.80 (0.44–1.46)
**Missing**	5/3151	2.08 (0.45–9.61)	5/3151	1.97 (0.48–8.14)	3/3153	-	1/3155	-
*P*_*trend*_		<0.0001		0.04		0.65		0.63
**Total body fat (%)**								
**<26.9**	120/147,125	Reference	40/147,205	Reference	50/147,195	Reference	35/147,210	Reference
**26.9–35.3**	150/154,061	1.54 (1.19–1.99)	40/154,171	0.64 (0.39–1.05)	60/154,151	1.30 (0.87–1.95)	30/154,181	0.72 (0.42–1.24)
**>35.3**	53/147,088	1.96 (1.30–2.96)	32/147,109	0.39 (0.21–0.72)	20/147,121	1.35 (0.67–2.73)	21/147,120	0.47 (0.22–1.00)
**Missing**	16/10,100	1.99 (1.08–3.68)	12/10,104	2.20 (1.03–4.69)	7/10,109	1.75 (0.62–4.92)	6/10,110	2.16 (0.81–5.74)
*P*_*trend*_		0.0006		0.01		0.43		0.14
**Trunk fat (%)**								
**<27.7**	84/147,724	Reference	43/147,765	Reference	44/147,764	Reference	33/147,775	Reference
**27.7–34.5**	135/153,201	1.62 (1.22–2.16)	41/153,295	0.76 (0.49–1.19)	50/153,286	1.05 (0.68–1.61)	32/153,304	0.79 (0.47–1.31)
**>34.5**	105/147,333	2.34 (1.70–3.22)	29/147,409	0.43 (0.25–0.74)	36/147,402	1.34 (0.81–2.21)	20/147,418	0.50 (0.27–0.94)
**Missing**	15/10,116	2.14 (0.12–4.09)	11/10,120	2.02 (0.93–4.37)	7/10,124	1.63 (0.58–4.61)	7/10,124	2.58 (0.28–0.97)
*P*_*trend*_		<0.0001		0.009		0.48		0.09
BMI (kg/m^2^)^c^^,^^e^								
Underweight^d^	1/2326	-	4/2323	-	1/2326		2/2325	
**Normal weight**	51/147,311	Reference	45/147,317	Reference	30/147,332	Reference	28/147,334	Reference
**Overweight**	153/193,676	1.31 (0.86–1.99)	54/193,775	1.31 (0.75–2.29)	60/193,769	0.98 (0.53–1.80)	38/193,791	0.78 (0.40–1.51)
**Obese**	128/111,520	1.68 (0.98–2.89)	16/111,632	0.95 (0.36–2.47)	42/111,606	0.86 (0.37–1.99)	23/111,625	0.98 (0.37–2.61)
**Missing**	6/3541	1.27 (0.17–9.56)	5/3542	3.39 (0.61–19.01)	4/3543	2.16 (0.22–21.69)	1/3546	-
*P*_*trend*_		0.16		0.44		0.91		0.63

BMI, body mass index; CI, confidence interval; HR, hazard ratio

^a^Stratified on age (5 year categories), sex, Townsend deprivation index (quintiles), recruitment assessment centre and adjusted for smoking status and education

^b^Stratified on age (5 year categories), sex, Townsend deprivation index (quintiles), recruitment assessment centre and adjusted for smoking status, education and alcohol intake

^c^Underweight (BMI<18.5), normal weight (18.5≤BMI<25), overweight (25≤BMI<30) and obese (BMI ≥30)

^d^We excluded underweight group from the analysis due to few numbers of cases

^e^Model additionally adjusted for waist circumference, hip circumference, body fat and trunk fat percentage

For tests of linear trend, the missing category was excluded

Interaction analyses by sex were not statistically significant (all p-interaction values >0.05) except for hip circumference and oesophageal squamous cell carcinoma (p-interaction = 0.02); however, we conducted analyses stratified by sex in order to allow comparisons of the anthropometric variables in women with the reproductive variables that were only available among women.

We found weight, BMI, hip circumference, waist circumference, waist-to-hip ratio, waist-to-height ratio, total percent body fat and trunk fat percentage were all statistically significantly positively associated with oesophageal adenocarcinoma in men (Table 2). While in women, only waist-to-height ratio was positively associated with oesophageal adenocarcinoma (HR 3.87, 95% CI: 1.31–11.46 for >0.55 vs <0.48) (Table 3).

**Table 2:** Adjusted hazard ratios (HR) and 95% confidence intervals (CI) for oesophageal and gastric cancer by subtype and subsite in men (n = 214,293) according to anthropometric factors in the UK Biobank cohort.

** **	** **		**Oesophageal cancer**			**Gastric cancer**		** **
	Adenocarcinoma		Squamous cell carcinoma		Cardia		Non-cardia	
** Men**^a^	Cases/Non-cases	Adjusted HR^b^ (95% CI)	Cases/Non-cases	Adjusted HR^c^ (95% CI)	Case/Non-cases	Adjusted HR^b^ (95% CI)	Cases/Non-cases	Adjusted HR^b^ (95% CI)
**Height (cm)**								
**<173**	130/69,240	Reference	28/69,342	Reference	42/69,328	Reference	20/69,350	Reference
**173–179**	97/82,976	0.76 (0.58–1.00)	13/83,060	0.47 (0.24–0.93)	44/83,029	1.04 (0.66–1.63)	24/83,049	1.33 (0.71–2.49)
**>179**	60/60,154	0.77 (0.55–1.07)	10/60,204	0.57 (0.26–1.23)	24/60,190	1.03 (0.61–1.75)	13/60,201	1.35 (0.65–2.83)
**Missing**	4/1632	1.01 (0.22–4.56)	3/1633	2.03 (0.26–15.6)	3/1633	-	0/1636	-
*P*_*trend*_		0.11		0.07		0.99		0.62
**Weight (kg)**								
**<78**	78/69,674	Reference	22/69,730	Reference	26/69,726	Reference	21/69,731	Reference
**78–90**	90/72,697	1.10 (0.80–1.52)	16/72,771	0.83 (0.43–1.60)	38/72,749	1.60 (0.95–2.69)	20/72,767	0.86 (0.46–1.62)
**>90**	118/699,83	1.63 (1.20–2.20)	13/70,088	0.64 (0.31–1.32)	45/70,056	1.83 (1.08–3.07)	16/70,085	0.70 (0.36–1.37)
**Missing**	5/1648	2.03 (0.58–7.09)	3/1650	2.22 (0.29–17.27)	4/1649	2.77 (0.36–20.9)	0/1653	-
*P*_*trend*_		0.002		0.48		0.07		0.58
BMI (kg/m^2^) ^d^								
Underweight^e^	1/498	-	1/498	-	1/498	-	2/497	-
**Normal weight**	40/52,874	Reference	12/52,902	Reference	18/52,896	Reference	14/52,900	Reference
**Overweight**	132/104,752	1.50 (1.03–2.18)	30/104,854	1.44 (0.71–2.90)	52/104,832	1.44 (0.81–2.54)	27/104,857	0.78 (0.40–1.51)
**Obese**	113/54,009	2.39 (1.63–3.52)	8/54,114	0.63 (0.24–1.64)	38/54,084	1.83 (1.00–3.37)	14/54,108	0.68 (0.31–1.47)
**Missing**	5/1869	2.42 (0.69–8.51)	3/1871	2.64 (0.33–21.39)	4/1870	2.61 (0.34–20.05)	0/1874	-
*P*_*trend*_		<0.0001		0.12		0.15		0.60
**Hip circumference (cm)**								
**<100**	70/64,437	Reference	20/64,487	Reference	27/64,480	Reference	23/64,484	Reference
**100–106**	108/86,038	1.13 (0.83–1.55)	18/86,128	0.79 (0.41–1.52)	41/86,105	1.21 (0.73–1.99)	22/86,124	0.70 (0.38–1.28)
**>106**	109/62,164	1.59 (1.16–2.18)	14/62,259	0.77 (0.38–1.57)	42/62,231	1.49 (0.89–2.50)	12/62,261	0.49 (0.24–1.03)
**Missing**	4/1363	1.77 (0.37–8.39)	2/1365	-	3/1364	-	0/1367	-
*P*_*trend*_		0.008		0.70		0.31		0.15
**Waist circumference (cm)**								
**<92**	51/69,908	Reference	15/69,944	Reference	20/699,39	Reference	17/69,942	Reference
**92–101**	101/77,991	1.53 (1.08–2.19)	26/78,066	1.60 (0.83–3.11)	39/78,053	1.47 (0.83–2.59)	22/78,070	0.91 (0.47–1.75)
**>101**	135/64,779	2.23 (1.58–3.14)	11/64,903	0.68 (0.30–1.56)	51/64,863	2.21 (1.27–3.84)	18/64,896	0.78 (0.39–1.55)
**Missing**	4/1324	2.41 (0.50–11.56)	2/1326	-	3/1325	-	0/1328	-
*P*_*trend*_		<0.0001		0.06		0.02		0.77
**Waist to hip ratio**								
**<0.91**	52/68,330	Reference	16/68,366	Reference	20/68,362	Reference	14/68,368	Reference
**0.91–0.96**	79/79,362	1.02 (0.70–1.48)	21/79,420	1.06 (0.54–2.09)	35/79,406	1.11 (0.62–1.98)	21/79,420	0.97 (0.48–1.95)
**>0.96**	156/64,937	2.11 (1.51–2.96)	15/65,078	0.73 (0.34–1.56)	55/65,038	1.92 (1.11–3.29)	22/65,071	0.95 (0.47–1.92)
**Missing**	4/1373	2.03 (0.43–9.66)	2/1375	-	3/1374	-	0/1377	-
*P*_*trend*_		<0.0001		0.55		0.02		0.99
**Waist to height ratio**								
**<0.52**	36/62,410	Reference	15/62,431	Reference	20/62,426	Reference	15/62,431	Reference
**0.52–0.57**	100/81,254	1.63 (1.09–2.44)	19/81,335	0.94 (0.46–1.90)	37/81,317	1.16 (0.65–2.05)	22/81,332	0.81 (0.41–1.61)
**>0.57**	151/68,674	2.58 (1.75–3.80)	17/68,808	0.81 (0.38–1.70)	53/68,772	1.69 (0.97–2.95)	20/68,805	0.69 (0.34–1.41)
**Missing**	4/1664	2.14 (0.46–9.96)	3/1665	2.49 (0.31–19.91)	3/1665	-	0/1668	-
*P*_*trend*_		<0.0001		0.84		0.10		0.60
**Total body fat (%)**								
**<22.9**	41/68,393	Reference	12/68,422	Reference	24/68,410	Reference	18/68416	Reference
**22.9–27.8**	95/72,250	1.93 (1.30–2.86)	18/72,597	1.46 (0.68–3.13)	34/72,581	1.10 (0.64–1.88)	17/72598	0.66 (0.33–1.32)
**>27.8**	140/67,888	2.57 (1.75–3.76)	17/68,011	1.07 (0.48–2.38)	48/67,980	1.29 (0.76–2.18)	17/68011	0.57 (0.28–1.15)
**Missing**	15/5201	3.32 (1.70–6.51)	7/5201	4.09 (1.34–12.55)	7/5209	2.04 (0.70–5.99)	5/5211	3.06 (1.09–8.58)
*P*_*trend*_		<0.0001		0.54		0.61		0.26
**Trunk fat (%)**								
**<25.2**	44/68,928	Reference	12/68,960	Reference	26/68,946	Reference	18/68,954	Reference
**25.2–30.7**	96/71,807	1.98 (1.35–2.90)	19/71,884	1.59 (0.75–3.38)	31/71,872	0.96 (0.56–1.64)	15/71,888	0.69 (0.34–1.40)
**>30.7**	137/68,187	2.41 (1.66–3.49)	17/68,307	1.08 (0.49–2.41)	49/68,275	1.25 (0.75–2.09)	18/68,306	0.67 (0.33–1.36)
**Missing**	14/5080	2.98 (1.49–5.94)	6/5088	3.13 (0.94–10.41)	7/5087	1.92 (0.66–5.59)	6/5088	4.06 (1.54–10.69)
*P*_*trend*_		<0.0001		0.38		0.49		0.46

BMI, body mass index; CI, confidence interval; HR, hazard ratio

^a^Sex-specific tertiles were used in the analyses except for BMI

^b^Stratified on age (5 year categories), Townsend deprivation index (quintiles), recruitment assessment centre and adjusted for smoking status and education

^c^Stratified on age (5 year categories), Townsend deprivation index (quintiles), recruitment assessment centre and adjusted for smoking status, education and alcohol intake

^d^Underweight (BMI<18.5), normal weight (18.5≤BMI<25), overweight (25≤BMI<30) and obese (BMI ≥30)

^e^We excluded underweight group from the analysis due to few numbers of cases

For tests of linear trend, the missing category was excluded

**Table 3:** Adjusted hazard ratios (HR) and 95% confidence intervals (CI) for oesophageal and gastric cancer by subtype and subsite in women (n = 244,420) according to anthropometric factors in the UK Biobank cohort.

** **	** **		**Oesophageal cancer**			**Gastric cancer**		** **
	Adenocarcinoma		Squamous cell carcinoma		Cardia		Non-cardia	
** Women**^a^	Cases/Non-cases	Adjusted HR^b^ (95% CI)	Cases/Non-cases	Adjusted HR^c^ (95% CI)	Cases/Non-cases	Adjusted HR^b^ (95% CI)	Cases/Non-cases	Adjusted HR^b^ (95% CI)
**Height (cm)**								
**<160**	13/78,011	Reference	21/78,003	Reference	6/78,018	Reference	15/78,009	Reference
**160–165**	23/89,517	1.79 (0.90–3.57)	33/89,507	1.46 (0.82–2.59)	10/89,530	1.40 (0.48–4.13)	10/89,530	0.70 (0.31–1.57)
**>165**	11/75,433	1.21 (0.53–2.74)	14/75,430	0.84 (0.41–1.76)	8/75,436	2.31 (0.77–6.95)	9/75,435	0.88 (0.36–2.15)
**Missing**	1/1411	-	2/1410	3.23 (0.42–24.83)	0/1412	-	1/1411	-
*P*_*trend*_		0.21		0.21		0.32		0.69
**Weight (kg)**								
**<64**	9/79,250	Reference	38/79,221	Reference	8/79,251	Reference	12/79,247	Reference
**64–75**	18/83,683	1.92 (0.86–4.28)	19/83,682	0.41 (0.22–0.74)	7/83,694	1.01 (0.33–3.03)	11/83,690	0.84 (0.37–1.92)
**>75**	20/79,853	2.11 (0.95–4.67)	11/79,862	0.28 (0.14–0.57)	9/79,864	1.20 (0.42–3.47)	11/79,862	0.79 (0.34–1.85)
**Missing**	1/1586	-	2/1585	1.39 (0.19–10.39)	0/1587	-	1/1586	-
*P*_*trend*_		0.17		0.0002		0.92		0.85
BMI (kg/m^2^)^d^								
Underweight^e^	0/1828	-	3/1825	-	0/1828	-	0/1828	-
**Normal weight**	11/94,437	Reference	33/94,415	Reference	12/94,436	Reference	14/94,434	Reference
**Overweight**	21/88,924	1.82 (0.87–3.80)	24/88,921	0.65 (0.37–1.14)	8/88,937	0.58 (0.22–1.52)	11/88,934	0.64 (0.28–1.46)
**Obese**	15/57,511	1.95 (0.88–4.32)	8/57,518	0.34 (0.15–0.79)	4/57,522	0.41 (0.12–1.35)	9/57,517	0.84 (0.36–1.97)
**Missing**	1/1672	-	2/1671	1.65 (0.22–12.41)	0/1673	-	1/1672	-
*P*_*trend*_		0.20		0.03		0.29		0.57
**Hip circumference (cm)**								
**<98**	9/73,708	Reference	35/73,682	Reference	8/73,709	Reference	14/73,703	Reference
**98–106**	21/93,821	1.74 (0.79–3.81)	28/93,814	0.58 (0.34–0.99)	9/93,833	0.89 (0.31–2.56)	10/93,832	0.53 (0.24–1.20)
**>106**	17/75,486	1.63 (0.72–3.69)	6/75,497	0.15 (0.06–0.38)	7/75,496	0.77 (0.26–2.42)	10/75,493	0.55 (0.24–1.29)
**Missing**	1/1375	-	1/1375	-	0/1358	-	1/1375	-
*P*_*trend*_		0.37		0.0002		0.92		0.23
**Waist circumference (cm)**								
**<78**	6/77,725	Reference	32/77,699	Reference	9/77,722	Reference	11/77,720	Reference
**78–89**	22/89,834	2.66 (1.07–6.58)	23/89,833	0.53 (0.30–0.94)	8/89,848	0.73 (0.26–2.07)	13/89,843	0.79 (0.35–1.79)
**>89**	19/75,469	2.42 (0.96–6.15)	14/75,474	0.38 (0.19–0.74)	7/75,481	0.60 (0.19–1.80)	10/75,478	0.71 (0.30–1.69)
**Missing**	1/1344	-	1/1344	-	0/1345	-	1/1344	-
*P*_*trend*_		0.10		0.008		0.65		0.72
**Waist to hip ratio**								
**<0.78**	4/70,458	Reference	17/70,445	Reference	7/70,455	Reference	10/70,452	Reference
**0.78–0.85**	29/103,163	3.73 (1.30–10.66)	28/103,164	0.96 (0.51–1.81)	9/103,183	0.91 (0.29–2.83)	12/103,180	0.79 (0.33–1.89)
**>0.85**	14/69,372	2.48 (0.80–7.63)	24/69,362	1.08 (0.56–2.09)	8/69,378	1.02 (0.31–3.29)	12/69,374	1.01 (0.42–2.44)
**Missing**	1/1379	-	1/1379	-	0/1380	-	1/1379	-
*P*_*trend*_		0.04		0.92		0.97		0.80
**Waist to height ratio**								
**<0.48**	4/75,530	Reference	26/75,508	Reference	6/75,528	Reference	11/75,523	Reference
**0.48–0.55**	22/93,915	3.50 (1.20–10.22)	30/93,907	0.73 (0.42–1.27)	12/93,925	1.86 (0.58–5.93)	13/93,924	0.78 (0.34–1.79)
**>0.55**	21/73,440	3.87 (1.31–11.46)	12/73,449	0.36 (0.17–0.74)	6/73,455	0.84 (0.22–3.17)	10/73,451	0.67 (0.27–1.66)
**Missing**	1/1487	-	2/1486	-	0/1488	-	1/1487	-
*P*_*trend*_		0.05		0.02		0.25		0.68
**Total body fat (%)**								
**<33.7**	12/78,623	Reference	29/78,606	Reference	9/78,626	Reference	12/78,623	Reference
**33.7–39.8**	10/82,586	0.68 (0.29–1.57)	25/82,571	0.65 (0.36–1.15)	7/82,589	0.69 (0.24–2.02)	12/82,584	0.72 (0.31–1.64)
**>39.8**	25/78,264	1.61 (0.79–3.26)	11/78,278	0.32 (0.15–0.66)	8/78,281	0.73 (0.25–2.11)	10/78,279	0.60 (0.25–1.41)
**Missing**	1/4899	-	5/4895	2.13 (0.73–13.72)	0/4900	-	1/4899	-
*P*_*trend*_		0.06		0.009		0.77		0.49
**Trunk fat (%)**								
**<31.0**	14/78,667	Reference	30/78,651	Reference	10/78671	Reference	13/78,668	Reference
**31.0–37.8**	9/82,179	0.52 (0.23–1.21)	22/82,166	0.58 (0.33–1.05)	6/82182	0.53 (0.18–1.58)	11/82,177	0.61 (0.27–1.39)
**>37.8**	24/78,490	1.32 (0.67–2.61)	13/78,501	0.35 (0.17–0.71)	8/78506	0.74 (0.27–2.05)	10/78,504	0.58 (0.25–1.35)
**Missing**	1/5036	-	5/5032	2.06 (0.71–6.00)	0/5037	-	1/5036	-
*P*_*trend*_		0.06		0.01		0.52		0.35

BMI, body mass index; CI, confidence interval; HR, hazard ratio

^a^Sex-specific tertiles were used in the analyses except for BMI

^b^Stratified on age (5 year categories), Townsend deprivation index (quintiles), recruitment assessment centre, and adjusted for smoking status, and education

^c^Stratified on age (5 year categories), Townsend deprivation index (quintiles), recruitment assessment centre, and adjusted for smoking status, education and alcohol intake

^d^Underweight (BMI<18.5), normal weight (18.5≤BMI<25), overweight (25≤BMI<30) and obese (BMI ≥30)

^e^ We excluded underweight group from the analysis due to few number of cases

For tests of linear trend, the missing category was excluded

For oesophageal squamous cell carcinoma, no statistically significant associations were observed with anthropometric measurements or body fat composition in men (Table 2). While in women, weight, BMI, hip circumference, waist circumference, waist-to-height ratio, body fat and trunk fat percentage were all statistically significantly inversely associated with oesophageal squamous cell carcinoma (Table 3).

For gastric cardia cancer, there were no statistically significant associations for anthropometric measures among women (Table 3). Among men, however, we observed positive associations for weight (HR 1.83, 95% CI: 1.08–3.07 for >90 vs <78 kg), BMI (HR 1.83, 95% CI: 1.00–3.37 for obese vs normal weight), waist circumference (HR 2.21, 95% CI: 1.27–3.84 for >101 vs <92 cm), and waist-to-hip ratio (HR 1.92, 95% CI:1.11–3.29 for >0.96 vs <0.91) and gastric cardia cancer; while there were no statistically significant associations for body fat or trunk fat (Table 2). For gastric non-cardia cancer, no statistically significant associations were observed with anthropometric measurements or body fat composition in either men or women (Tables 2 and 3).

BMI and waist-to-hip ratio were moderately correlated (r = 0.43), as were waist circumference and hip circumference (r = 0.74) in this dataset. To investigate abdominal obesity specifically, BMI and waist-to-hip ratio were mutually adjusted. The positive associations observed for oesophageal adenocarcinoma with BMI, waist circumference, waist-to-hip ratio in men were attenuated but remained statistically significant after adjustment for waist-to-hip ratio, hip circumference, and BMI, respectively (Table 4). While the positive association observed between hip circumference and oesophageal adenocarcinoma in men was no longer significant after adjustment for waist circumference (HR 0.84, 95% CI: 0.54–1.31 for >106 vs <100 cm) (Table 4). In addition, positive associations observed for oesophageal adenocarcinoma with total percent body fat and trunk fat percentage in men remained statistically significant after adjustment for BMI, waist circumference and hip circumference, separately (Table 4).

**Table 4:** Adjusted hazard ratios (HR) and 95% confidence intervals (CI) for oesophageal and gastric cancer by subtype and subsite in men (n = 214,293) according to anthropometric factors in the UK Biobank cohort (mutually adjusted).

			Oesophageal cancer			Gastric cancer		
	Adenocarcinoma		Squamous cell carcinoma		Cardia		Non-cardia	
Men^a^	Cases/Non-cases	Adjusted HR^b^ (95% CI)	Cases/Non-cases	Adjusted HR^c^ (95% CI)	Cases/Non-cases	Adjusted HR^b^ (95% CI)	Cases/Non-cases	Adjusted HR^b^ (95% CI)
BMI (kg/m^2^) ^**d**^ adjusted for waist to hip ratio								
Underweight^**e**^	1/498	-	1/498	-	1/498	-	2/497	-
**Normal weight**	40/52,874	Reference	12/52,902	Reference	18/52,896	Reference	14/52,900	Reference
**Overweight**	132/104,752	1.32 (0.89–1.97)	30/104,854	1.47 (0.69–3.09)	52/104,832	1.25 (0.68–2.29)	27/104,857	0.68 (0.33–1.40)
**Obese**	113/54,009	1.75 (1.13–2.72)	8/54,114	0.67 (0.23–1.92)	38/54,084	1.34 (0.68–2.67)	14/54,108	0.55 (0.22–1.34)
**Missing**	5/1869	1.97 (0.29–13.42)	3/1871	10.29 (1.20–88.25)	4/1870	4.66 (0.58–37.17)	0/1874	-
*P*_*trend*_		0.03		0.15		0.70		0.41
**Waist circumference (cm) adjusted for hip circumference**								
**<92**	51/69,908	Reference	15/69,944	Reference	20/69,939	Reference	17/69,942	Reference
**92–101**	101/77,991	1.70 (1.15–2.53)	26/78,066	1.80 (0.85–3.81)	39/78,053	1.54 (0.82–2.91)	22/78,070	1.21 (0.59–2.48)
**>101**	135/64,779	2.53 (1.59–4.04)	11/64,903	0.66 (0.22–2.02)	51/64,863	2.60 (1.24–5.49)	18/64,896	1.53 (0.60–3.90)
**Missing**	4/1324	-	2/1326	-	3/1325	-	0/1328	-
*P*_*trend*_		0.0005		0.04		0.03		0.67
**Hip circumference (cm) adjusted for waist circumference**								
**<100**	70/64,437	Reference	20/64,487	Reference	27/64,480	Reference	23/64,484	Reference
**100–106**	108/86,038	0.79 (0.55–1.14)	18/86,128	0.70 (0.34–1.45)	41/86,105	0.85 (0.48–1.52)	22/86,124	0.60 (0.30–1.21)
**>106**	109/62,164	0.84 (0.54–1.31)	14/62,259	1.04 (0.40–2.74)	42/62,231	0.75 (0.37–1.54)	12/62,261	0.36 (0.14–0.98)
**Missing**	4/1363	-	2/1365	-	3/1364	-	0/1367	-
*P*_*trend*_		0.46		0.48		0.74		0.13
**Waist to hip ratio adjusted for BMI**								
**<0.91**	52/68,330	Reference	16/68,366	Reference	20/68,362	Reference	14/68,368	Reference
**0.91–0.96**	79/79,362	0.91 (0.62–1.34)	21/79,420	1.05 (0.51–2.17)	35/79,406	1.07 (0.58–1.98)	21/79,420	1.29 (0.61–2.75)
**>0.96**	156/64,937	1.66 (1.13–2.44)	15/65,078	0.85 (0.37–1.99)	55/65,038	1.74 (0.93–3.25)	22/65,071	1.46 (0.62–3.40)
**Missing**	4/1373	1.22 (0.11–13.19)	2/1375	-	3/1375	-	0/1377	-
*P*_*trend*_		0.0002		0.85		0.08		0.68
**Total body fat (%) adjusted for BMI**								
**<22.9**	41/68,393	Reference	12/68,422	Reference	24/68,410	Reference	18/68,416	Reference
**22.9–27.8**	95/72,250	1.80 (1.16–2.79)	18/72,597	1.39 (0.59–3.23)	34/72,581	0.95 (0.52–1.75)	17/72,598	0.77 (0.35–1.68)
**>27.8**	140/67,888	2.03 (1.25–3.30)	17/68,011	1.37 (0.51–3.67)	48/67,980	0.96 (0.48–1.93)	17/68,011	0.73 (0.28–1.91)
**Missing**	15/5201	2.09 (1.36–6.17)	7/5209	5.26 (1.52–18.17)	7/5209	1.54 (0.45–5.33)	5/5211	4.00 (1.32–12.13)
*P*_*trend*_		0.01		0.74		0.99		0.76
**Trunk fat (%) adjusted for BMI**								
**<25.2**	44/68,928	Reference	12/68,960	Reference	26/68,946	Reference	18/68,954	Reference
**25.2–30.7**	96/71,807	1.81 (1.19–2.76)	19/71,884	1.72 (0.74–3.99)	31/71,872	0.84 (0.46–1.51)	15/71,888	0.80 (0.36–1.76)
**>30.7**	137/68,187	1.83 (1.15–2.92)	17/68,307	1.59 (0.60–4.23)	49/68,275	0.96 (0.50–1.85)	18/68,306	0.89 (0.36–2.27)
**Missing**	14/5080	2.50 (1.14–5.46)	6/5088	3.88 (0.99–15.18)	7/5087	1.55 (0.45–5.30)	6/5088	6.01 (2.13–16.95)
*P*_*trend*_		0.02		0.44		0.79		0.85
**Total body fat (%) adjusted for waist circumference**								
**<22.9**	41/68,393	Reference	12/68,422	Reference	24/68,410	Reference	18/68,416	Reference
**22.9–27.8**	95/72,250	1.72 (1.12–2.64)	18/72,597	1.27 (0.55–2.93)	34/72,581	0.79 (0.43–1.46)	17/72,598	0.57 (0.26–1.24)
**>27.8**	140/67,888	2.01 (1.25–3.23)	17/68,011	1.21 (0.46–3.24)	48/67,980	0.64 (0.31–1.29)	17/68,011	0.45 (0.17–1.18)
**Missing**	15/5201	2.79 (1.34–5.83)	7/5209	5.93 (1.84–19.16)	7/5209	1.45 (0.47–4.51)	5/5211	3.44 (1.12–10.54)
*P*_*trend*_		0.02		0.85		0.48		0.23
**Trunk fat (%) adjusted for waist circumference**								
**<25.2**	44/68,928	Reference	12/68,960	Reference	26/68,946	Reference	18/68,954	Reference
**25.2–30.7**	96/71,807	1.73 (1.41–2.61)	19/71,884	1.49 (0.66–3.35)	31/71,872	0.69 (0.38–1.26)	15/71,888	0.61 (0.28–1.34)
**>30.7**	137/68,187	1.81 (1.14–2.86)	17/68,307	1.35 (0.52–3.51)	49/68,275	0.68 (0.35–1.33)	18/68,306	0.58 (0.23–1.46)
**Missing**	14/5080	2.39 (1.12–5.10)	6/5088	4.52 (1.30–15.79)	7/5087	1.43 (0.47–4.38)	6/5088	5.04 (1.77–14.36)
*P*_*trend*_		0.02		0.63		0.43		0.40
**Total body fat (%) adjusted for hip circumference**								
**<22.9**	41/68,393	Reference	12/68,422	Reference	24/68,410	Reference	18/68,416	Reference
**22.9–27.8**	95/72,250	1.96 (1.30–2.95)	18/72,597	1.51 (0.68–3.34)	34/72,581	0.99 (0.57–1.74)	17/72,598	0.74 (0.36–1.52)
**>27.8**	140/67,888	2.52 (1.63–3.89)	17/68,011	1.15 (0.45–2.94)	48/67,980	1.03 (0.55–1.92)	17/68,011	0.71 (0.31–1.66)
**Missing**	15/5201	3.34 (1.62–6.87)	7/5209	5.89 (1.83–18.95)	7/5209	2.09 (0.69–6.31)	5/5211	4.57 (1.57–13.27)
*P*_*trend*_		0.0002		0.54		0.99		0.65
**Trunk fat (%) adjusted for hip circumference**								
**<25.2**	44/68,928	Reference	12/68,960	Reference	26/68,946	Reference	18/68,954	Reference
**25.2–30.7**	96/71,807	1.99 (1.34–2.95)	19/71,884	1.67 (0.77–3.65)	31/71,872	0.87 (0.50–1.52)	15/71,888	0.77 (0.36–1.62)
**>30.7**	137/68,187	2.30 (1.50–3.51)	17/68,307	1.20 (0.48–3.02)	49/68,275	1.01 (0.56–1.85)	18/68,306	0.86 (0.37–1.98)
**Missing**	14/5080	2.89 (1.37–6.11)	6/5088	4.42 (1.27–15.39)	7/5087	1.99 (0.67–5.93)	6/5088	6.23 (2.29–16.91)
*P*_*trend*_		0.0004		0.37		0.81		0.78

BMI, body mass index; CI, confidence interval; HR, hazard ratio

^a^Sex-specific tertiles were used in the analyses except for BMI

^b^Stratified on age (5 year categories), Townsend deprivation index (quintiles), recruitment assessment centre and adjusted for smoking status, and education

^c^Stratified on age (5 year categories), Townsend deprivation index (quintiles), recruitment assessment centre and adjusted for smoking status, education and alcohol intake

^d^Underweight (BMI<18.5), normal weight (18.5≤BMI<25), overweight (25≤BMI<30) and obese (BMI ≥30)

^e^We excluded underweight group from the analysis due to few number of cases

For tests of linear trend, the missing category was excluded

For oesophageal squamous cell carcinoma, the inverse associations observed with BMI and hip circumference in women remained statistically significant after adjustment for waist-to-hip ratio and waist circumference, respectively (Table 5). While the inverse association observed between waist circumference and oesophageal squamous cell carcinoma in women was no longer significant after adjustment for hip circumference (HR 1.22, 95% CI: 0.52–2.87 for waist circumference >89 vs <78 cm) (Table 5).

**Table 5:** Adjusted hazard ratios (HR) and 95% confidence intervals (CI) for oesophageal and gastric cancer by subtype and subsite in women (n = 244,420) according to anthropometric factors in the UK Biobank cohort (mutually adjusted).

			Oesophageal cancer			Gastric cancer		
	Adenocarcinoma		Squamous cell carcinoma		Cardia		Non-cardia	
Women^a^	Cases/Non-cases	Adjusted HR^b^ (95% CI)	Cases/Non-cases	Adjusted HR^c^ (95% CI)	Cases/Non-cases	Adjusted HR^b^ (95% CI)	Cases/Non-cases	Adjusted HR^b^ (95% CI)
BMI (kg/m^2^) ^**d**^ adjusted for waist to hip ratio								
Underweight^**e**^	0/1828	-	3/1825	-	0/1828	-	0/1828	-
**Normal weight**	11/94,437	Reference	33/94,415	Reference	12/94,436	Reference	14/94,434	Reference
**Overweight**	21/88,924	1.65 (0.78–3.49)	24/88,921	0.58 (0.32–1.05)	8/88,937	0.52 (0.19–1.43)	11/88,934	0.62 (0.27–1.46)
**Obese**	15/57,511	1.80 (0.78–4.16)	8/57,518	0.28 (0.12–0.68)	4/57,522	0.34 (0.09–1.22)	9/57,517	0.77 (0.30–2.01)
**Missing**	1/1672	-	2/1671	4.86 (0.61–39.07)	0/1673	-	1/1672	-
*P*_*trend*_		0.33		0.01		0.21		0.55
**Waist circumference (cm) adjusted for hip circumference**								
**<78**	6/77,725	Reference	32/77,699	Reference	9/77,722	Reference	11/77,720	Reference
**78–89**	22/89,834	2.53 (0.94–6.83)	23/89,833	0.76 (0.41–1.42)	8/89,848	0.68 (0.21–2.21)	13/89,843	1.08 (0.43–2.71)
**>89**	19/75,469	2.44 (0.75–7.94)	14/75,474	1.22 (0.52–2.87)	7/75,481	0.51 (0.11–2.40)	10/75,478	1.18 (0.34–4.06)
**Missing**	1/1344	-	1/1344	-	0/1345	-	1/1344	-
*P*_*trend*_		0.19		0.42		0.69		0.97
**Hip circumference (cm) adjusted for waist circumference**								
**<98**	9/73,708	Reference	35/73,682	Reference	8/73,709	Reference	14/73,703	Reference
**98–106**	21/93,821	1.18 (0.49–2.82)	28/93,814	0.59 (0.32–1.10)	9/93,833	1.11 (0.34–3.64)	10/93,832	0.50 (0.20–1.27)
**>106**	17/75,486	1.00 (0.35–2.89)	6/75,497	0.12 (0.04–0.39)	7/75,496	1.26 (0.26–6.05)	10/75,493	0.49 (0.15–1.66)
**Missing**	1/1357	-	1/1357	-	0/1358	-	1/1357	-
*P*_*trend*_		0.87		0.002		0.96		0.32
**Waist to hip ratio adjusted for BMI**								
**<0.78**	4/70,458	Reference	17/70,445	Reference	7/70,455	Reference	10/70,452	Reference
**0.78–0.85**	29/103,163	3.14 (1.08–9.13)	28/103,164	1.33 (0.68–2.58)	9/103,183	1.14 (0.36–3.63)	12/103,180	0.85 (0.35–2.06)
**>0.85**	14/69,372	1.85 (0.57–5.99)	24/69,362	2.03 (0.97–4.23)	8/69,378	1.62 (0.45–5.81)	12/69,374	1.15 (0.43–3.07)
**Missing**	1/1379	-	1/1379	-	0/1380	-	1/1379	-
*P*_*trend*_		0.05		0.15		0.72		0.77
**Total body fat (%) adjusted for BMI**								
**<33.7**	12/78,623	Reference	29/78,606	Reference	9/78,626	Reference	12/78,623	Reference
**33.7–39.8**	10/82,586	0.42 (0.15–1.16)	25/82,571	0.75 (0.38–1.47)	7/82,589	1.02 (0.31–3.32)	12/82,584	0.69 (0.25–1.89)
**>39.8**	25/78,264	0.94 (0.32–2.80)	11/78,278	0.48 (0.17–1.41)	8/78,281	2.07 (0.44–9.71)	10/78,279	0.41 (0.10–1.69)
**Missing**	1/4899	-	5/4895	3.01 (0.84–10.83)	0/4900	-	1/4899	-
*P*_*trend*_		0.09		0.40		0.53		0.47
**Trunk fat (%) adjusted for BMI**								
**<31.0**	14/78,667	Reference	30/78,651	Reference	10/78671	Reference	13/78,668	Reference
**31.0–37.8**	9/82,179	0.34 (0.13–0.87)	22/82,166	0.69 (0.36–1.33)	6/82182	0.74 (0.23–2.39)	11/82,177	0.58 (0.22–1.49)
**>37.8**	24/78,490	0.74 (0.29–1.88)	13/78,501	0.55 (0.22–1.39)	8/78506	1.65 (0.42–6.56)	10/78,504	0.46 (0.14–1.54)
**Missing**	1/5036	-	5/5032	2.94 (0.84–10.29)	0/5037	-	1/5036	-
*P*_*trend*_		0.05		0.38		0.44		0.40
**Total body fat (%) adjusted for waist circumference**								
**<33.7**	12/78,623	Reference	29/78,606	Reference	9/78,626	Reference	12/78,623	Reference
**33.7–39.8**	10/82,586	0.44 (0.17–1.10)	25/82,571	0.79 (0.40–1.54)	7/82,589	0.84 (0.25–2.84)	12/82,584	0.71 (0.27–1.86)
**>39.8**	25/78,264	1.10 (0.43–2.84)	11/78,278	0.44 (0.16–1.22)	8/78,281	1.10 (0.24–5.11)	10/78,279	0.56 (0.16–1.98)
**Missing**	1/4899	-	5/4895	3.57 (1.12–11.38)	0/4900	-	1/4899	-
*P*_*trend*_		0.06		0.28		0.89		0.65
**Trunk fat (%) adjusted for waist circumference (cm)**								
**<31.0**	14/78,667	Reference	30/78,651	Reference	10/78671	Reference	13/78,668	Reference
**31.0–37.8**	9/82,179	0.34 (0.14–0.82)	22/82,166	0.71 (0.37–1.38)	6/82182	0.63 (0.19–2.11)	11/82,177	0.59 (0.23–1.52)
**>37.8**	24/78,490	0.82 (0.34–1.95)	13/78,501	0.51 (0.20–1.29)	8/78506	1.04 (0.25–4.25)	10/78,504	0.55 (0.17–1.73)
**Missing**	1/5036	-	5/5032	3.52 (1.13–10.93)	0/5037	-	1/5036	-
*P*_*trend*_		0.03		0.34		0.63		0.49
**Total body fat (%) adjusted for hip circumference**								
**<33.7**	12/78,623	Reference	29/78,606	Reference	9/78,626	Reference	12/78,623	Reference
**33.7–39.8**	10/82,586	0.56 (0.22–1.44)	25/82,571	0.95 (0.50–1.78)	7/82,589	0.68 (0.20–2.33)	12/82,584	0.95 (0.36–2.45)
**>39.8**	25/78,264	1.60 (0.59–4.36)	11/78,278	1.10 (0.43–2.81)	8/78,281	0.75 (0.16–3.45)	10/78,279	0.86 (0.23–3.25)
**Missing**	1/4899	-	5/4895	5.38 (1.78–16.20)	0/4900	-	1/4899	-
*P*_*trend*_		0.05		0.94		0.82		0.97
**Trunk fat (%) adjusted for hip circumference**								
**<31.0**	14/78,667	Reference	30/78,651	Reference	10/78671	Reference	13/78,668	Reference
**31.0–37.8**	9/82,179	0.40 (0.16–1.01)	22/82,166	0.85 (0.45–1.60)	6/82182	0.52 (0.15–1.76)	11/82,177	0.76 (0.30–1.96)
**>37.8**	24/78,490	1.07 (0.42–2.74)	13/78,501	1.11 (0.46–2.67)	8/78506	0.76 (0.18–3.21)	10/78,504	0.79 (0.23–2.73)
**Missing**	1/5036	-	5/5032	5.06 (1.69–15.12)	0/5037	-	1/5036	-
*P*_*trend*_		0.04		0.77		0.55		0.85

BMI, body mass index; CI, confidence interval; HR, hazard ratio

^a^Sex-specific tertiles were used in the analyses except for BMI

^b^Stratified on age (5 year categories), Townsend deprivation index (quintiles), recruitment assessment centre and adjusted for smoking status, and education

^c^Stratified on age (5 year categories), Townsend deprivation index (quintiles), recruitment assessment centre and adjusted for smoking status, education and alcohol intake

^d^Underweight (BMI<18.5), normal weight (18.5≤BMI<25), overweight (25≤BMI<30) and obese (BMI ≥30)

^e^We excluded underweight group from the analysis due to few number of cases

For tests of linear trend, the missing category was excluded

After mutual adjustment, BMI and waist-to-hip ratio were no longer statistically significantly associated with gastric cardia cancer in men (HR 1.34, 95% CI: 0.68–2.67 for obese vs normal weight and HR 1.74, 95% CI: 0.93–3.25 for waist-to-hip ratio >0.96 vs <0.91) (Table 4). While the positive association observed for gastric cardia cancer with waist circumference in men remained statistically significant after adjustment for hip circumference (Table 4). For gastric non-cardia cancer, results did not change in either men or women when we mutually adjusted for BMI and waist-to-hip ratio (Tables 4 and 5). While an inverse association was observed in men for gastric non-cardia cancer with hip circumference adjusted for waist circumference (HR 0.36, 95% CI: 0.14–0.98 for >106 vs <100 cm) (Table 4).

No statistically significant interactions were observed between anthropometric variables with BMI or smoking status and any of the outcomes.

### Reproductive factors

Baseline characteristics of women according to hormonal replacement therapy are presented in S2 Table. Women who reported using hormonal replacement therapy were older, were less educated and more likely to drink alcohol.

Table 6 presents the associations between reproductive factors and risk of oesophageal and gastric cancer by subtype and subsite, respectively. We found no statistically significant associations between reproductive factors and oesophageal adenocarcinoma, gastric cardia or non-cardia cancer. However, a positive association was observed for oesophageal squamous cell carcinoma for women who had ever had a still birth/miscarriage/termination (HR 1.84, 95% CI: 1.10–3.07). In addition, compared to women who had a younger age at first live birth (<25 years) and last live birth (<30 years), those with an older age at first live birth (≥25 years) and last live birth (≥30 years) had a decreased risk of oesophageal squamous cell carcinoma (HR 0.44, 95% CI: 0.22–0.88 and HR 0.44, 95% CI: 0.22–0.87, respectively) (Table 6).

**Table 6:** Adjusted hazard ratios (HR) and 95% confidence intervals (CI) for oesophageal and gastric cancer by subtype and subsite according to reproductive factors in women (n = 244,420) in the UK Biobank cohort.

** **	** **		**Oesophageal cancer**			**Gastric cancer**		** **
	Adenocarcinoma		Squamous cell carcinoma		Cardia		Non-cardia	
** **	Cases/Non-cases	Adjusted HR^a^ (95% CI)	Cases/Non-cases	Adjusted HR^b^ (95% CI)	Cases/Non-cases	Adjusted HR^a^ (95% CI)	Cases/Non-cases	Adjusted HR^a^ (95% CI)
**Age at menarche, years**								
<13	16/91,753	Reference	24/91,745	Reference	10/91,759	Reference	12/91,757	Reference
≥13	28/144,236	1.18 (0.63–2.20)	41/144,223	1.11 (0.64–1.92)	14/144,250	0.82 (0.35–1.96)	20/144,244	0.97 (0.47–2.02)
**Ever had still birth/miscarriage/termination**								
No	31/161,443	Reference	37/161,437	Reference	14/161,460	Reference	25/161,449	Reference
Yes	16/77,955	1.07 (0.58–1.97)	30/77,941	1.84 (1.10–3.07)	10/77,961	1.23 (0.51–2.99)	8/779,63	0.75 (0.34–1.69)
**Number of live births**								
<2	14/77,996	Reference	23/77,987	Reference	7/78003	Reference	9/78001	Reference
≥2	33/165,239	0.90 (0.48–1.71)	46/165,226	0.80 (0.47–1.36)	17/165255	0.95 (0.36–2.49)	25/165,247	1.23 (0.55–2.77)
**Age at first live birth, years**								
<25	14/73,815	Reference	31/73,798	Reference	9/73,820	Reference	16/73,813	Reference
≥25	19/91,061	1.75 (0.83–3.68)	15/91,065	0.44 (0.22–0.88)	8/91,072	1.18 (0.40–3.44)	8/91,072	0.56 (0.23–1.37)
**Age at last live birth, years**								
<30	19/75,249	Reference	33/75,235	Reference	11/75,257	Reference	15/75,253	Reference
≥30	14/89,282	0.84 (0.41–1.71)	13/89,283	0.44 (0.22–0.87)	6/89,290	0.68 (0.23–1.97)	9/89,287	0.71 (0.31–1.66)
**Ever used hormone replacement therapy**								
No	20/151,465	Reference	28/151,457	Reference	14/151,471	Reference	21/151,464	Reference
Yes	26/91,063	1.12 (0.61–2.06)	41/91,048	1.65 (0.96–2.85)	10/91,079	0.63 (0.26–1.52)	12/91,077	0.55 (0.27–1.17)

CI, confidence interval; HR, hazard ratio

^a^Stratified on age (5 year categories), Townsend deprivation index (quintiles), recruitment assessment centre and adjusted for smoking status, BMI, and education

^b^Stratified on age (5 year categories), Townsend deprivation index (quintiles), recruitment assessment centre and adjusted for smoking status, BMI, education and alcohol intake

The cases/non-cases may not sum to the total due to missing data

Our sensitivity analyses did not materially affect our findings. Specifically, a lag analysis excluding cases diagnosed in the first year of follow-up was conducted and models in which we applied tighter control for smoking by using a pack-years variable also did not alter our risk estimates meaningfully (results not shown).

## Discussion

In this large cohort of British adults, we found that both obesity and abdominal obesity specifically were positively associated with oesophageal adenocarcinoma and gastric cardia cancer, particularly in men. Furthermore, body fat composition was positively associated with oesophageal adenocarcinoma in men. Conversely, inverse associations were observed for oesophageal squamous cell carcinoma with obesity and body fat composition in women. No statistically significant associations were observed for gastric non-cardia cancer either with obesity or abdominal obesity or body fat composition in sex-stratified analyses. For reproductive factors in women and oesophageal squamous cell carcinoma, ever having a still birth/miscarriage/termination was positively associated with this malignancy and an older age at first live birth and last live birth was inversely associated.

Our study found a positive association between BMI and oesophageal adenocarcinoma among men, which is in line with previous cohort studies [32, 33], but we found no statistically significant association for women, which is inconsistent with data from the Million Women’s Study and the European Prospective Investigation into Cancer and Nutrition (EPIC) cohort [34, 35]. However, we had very few cases of oesophageal adenocarcinoma among women in this study (n = 48) and the test for interaction by sex was not statistically significant. After adjustment for waist-to-hip ratio, the positive association between BMI and oesophageal adenocarcinoma in men was attenuated but remained statistically significant. The NIH-AARP study and EPIC study showed a non-significant positive association between BMI and oesophageal adenocarcinoma adjusted for waist-to-hip ratio [35, 36]. Our study found a statistically significant positive association for gastric cardia cancer with BMI in men, but this association attenuated after adjustment for waist-to-hip ratio. The NIH-AARP study showed a significant positive association for gastric cardia cancer with BMI (HR 3.28, 95% CI: 1.76–6.11 for BMI ≥35 vs 18.5-<25) independently of waist-to-hip ratio [36], while the EPIC study showed no association between BMI and gastric cardia cancer [35]. In our study, analyses of body fat percentage and oesophageal adenocarcinoma revealed similar results to those observed for BMI, with only a positive association evident for oesophageal adenocarcinoma in men, while no statistically significant association was observed for gastric cardia cancer and body fat percentage in men or women. The Melbourne Collaborative Cohort Study examined body fat percentage and reported no significant association for lower oesophagus/gastric cardia combined [10].

In our study, waist-to-hip ratio and waist circumference were positively associated with oesophageal adenocarcinoma in men independently of BMI and hip circumference. Furthermore, a positive association was observed between hip circumference and oesophageal adenocarcinoma in men, but this association attenuated after adjustment for waist circumference. Recent findings from the EPIC study analysis reported positive associations for oesophageal adenocarcinoma with waist circumference, and waist-to-hip ratio in both men and women independently of hip circumference and BMI [35].

Waist circumference was positively associated with gastric cardia cancer in men independently of hip circumference in our analysis. In addition, waist-to-hip ratio was positively associated with gastric cardia cancer in men, but this association attenuated after adjustment for BMI. A meta-analysis of prospective cohort studies reported a significant positive association for gastric cardia cancer with waist circumference (RR 1.94, 95% CI: 1.30–2.91) but not with waist-to-hip ratio (RR 1.41, 95% CI: 0.79–2.51) [37]. A recent analysis in the EPIC study showed a significant positive association between waist circumference and gastric cardia cancer in men (HR 1.99, 95% CI: 1.10–3.59 for >98 vs <90 cm) when adjusted for hip circumference [35]. This cohort study also showed a positive association between waist-to-hip ratio and gastric cardia cancer in women independently of BMI [35], while the NIH-AARP study showed no association between waist-to-hip ratio and gastric cardia cancer [36].

Taken together, our findings show that both obesity and abdominal obesity specifically seems to be associated with oesophageal adenocarcinoma and gastric cardia cancer, particularly in men, although statistical power was limited for analyses in women. One proposed mechanism linking obesity with oesophageal adenocarcinoma and gastric cardia cancer is via gastro-oesophageal reflux disease, which is associated with an increased risk of oesophageal adenocarcinoma and gastric cardia cancer [36, 38]. Furthermore, it is evident that obesity can lead to higher levels of pro-inflammatory cytokines, insulin, leptin and sex steroids, which have all been associated with cancer at various anatomic sites [13]; in addition to these potential mechanisms, there may be other metabolic perturbations associated with obesity that affect cancer risk.

In our study, weight, BMI, hip circumference, waist circumference, waist-to-height ratio, body fat and trunk fat percentage were all inversely associated with oesophageal squamous cell carcinoma in women only. After adjustment for waist-to-hip ratio, the inverse association between BMI and oesophageal squamous cell carcinoma in women was attenuated but remained statistically significant; furthermore, mutual adjustment for waist and hip circumference attenuated the associations but remained statistically significant for hip circumference only. Two previous meta-analyses have reported an inverse association between BMI and oesophageal squamous cell carcinoma [2, 7]. The few cohort studies that examined the association between abdominal obesity and oesophageal squamous cell carcinoma showed inverse associations [35] or no associations [12, 39]. To our knowledge, no cohort study has examined body fat composition measurements in relation to oesophageal squamous cell carcinoma. The underlying mechanisms between adiposity and oesophageal squamous cell carcinoma are not well-known and needs further investigation but we cannot rule out residual confounding by smoking in some of the models because we had too few cases to conduct analyses stratified by smoking status.

Our study found no statistically significant associations between anthropometric variables and gastric non-cardia cancer, except an inverse association between hip circumference when it was adjusted for waist circumference and gastric non-cardia cancer in men. Recent findings from the EPIC study and previous meta-analyses showed no associations between anthropometric factors and gastric non-cardia cancer [35, 37, 40]. In our study, no statistically significant association was observed with body fat or trunk fat percentage and gastric non-cardia cancer, which is in line with findings from the Melbourne Collaborative Cohort Study [10].

In our study, several anthropometric measures (BMI, waist circumference, hip circumference, waist-to-hip ratio etc.) were assessed in relation to upper gastrointestinal cancers. Generally, obesity is measured using BMI but there are uncertainties about whether BMI captures enough information to understand the relevant biological mechanisms underlying the associations between adiposity and cancer risk [41]. Abdominal obesity, commonly measured using waist circumference, waist-to-hip ratio and waist-to-height ratio, may reveal additional associations. Furthermore, abdominal obesity may be a better predictor for cancer risk than BMI [37, 42], and is strongly associated with insulin resistance [43]. However, these anthropometric measures do not differentiate between lean and fat mass [44]; in this study, we were able to evaluate bioelectrical impedance measurements to assess total body fat and trunk fat percentage, which may more accurately reveal the association between body fat distribution and risk of upper gastrointestinal cancers.

For reproductive factors, a recent analysis in the EPIC study showed an inverse association between parity and oesophageal adenocarcinoma and between age at first pregnancy and gastric non-cardia cancer; whilst there was a positive association between bilateral ovariectomy and gastric non-cardia cancer [35]. Although we found no statistically significant associations between reproductive factors and oesophageal adenocarcinoma or gastric cardia or non-cardia cancer, we observed a positive association between stillbirth/miss-carriage/termination and oesophageal squamous cell carcinoma. In addition, inverse associations were observed for oesophageal squamous cell carcinoma with older age at first live birth and last live birth. A meta-analysis reported no significant association between abortion and oesophageal cancer, although this analysis did not stratify by oesophageal cancer subtypes [18]. A nested case-control study in Sweden showed a non-significant decreased risk for oesophageal squamous cell carcinoma in women who had first given birth at an older age (OR 0.61, 95% CI: 0.35–1.07 for ≥30 vs <20 years) [45] but the Million Women’s Health study showed no association [23]. In our study, we had few oesophageal and gastric cancer cases by subtype/subsite among women, where estimates for most of the reproductive factors may be underpowered to detect the modest associations observed in previous large cohort analyses [23, 35]. There are some proposed mechanisms to support an association between oestrogen and oesophageal cancer; for example, oestrogen receptors (ERα and ERβ) have been found in oesophageal carcinoma [46, 47], and treatment with selective oestrogen receptor ligands may inhibit cell growth and induce apoptosis [48, 49].

Strengths of our study include its prospective study design and availability of objectively measured anthropometric and body fat composition measurements. In addition, this study is one of the largest to examine bioelectrical impedance measurements to evaluate the association between body fat percentage and trunk fat percentage in relation to upper gastrointestinal cancers. Our study has some limitations. Even though we adjusted for several potential confounding variables, we lacked information on *Helicobacter Pylori* infection, which has been associated with gastric non-cardia cancer; hence, residual confounding cannot be excluded. We analysed anthropometric and reproductive factors in association with both oesophageal and gastric cancer by subtype and subsite; therefore, some of our associations may have arisen by chance as a consequence of multiple comparisons. Furthermore, despite the large size of the UK Biobank cohort, the number of cases in some analyses was quite small. Lastly, UK Biobank study participants are mainly of white British ancestry, which limits the generalizability of our findings to other ethnicities.

In conclusion, our findings for oesophageal adenocarcinoma, oesophageal squamous cell carcinoma and gastric non-cardia cancer largely agree with the data from the EPIC study [35], whereas the data for gastric cardia cancer was less consistent between the two large studies. We report that obesity and abdominal obesity specifically were positively associated with oesophageal adenocarcinoma and gastric cardia cancer, particularly in men. Furthermore, some reproductive factors in women may influence risk for oesophageal squamous cell carcinoma. Taken together, these results may support a role for hormonal pathways in the development of oesophageal and gastric cardia cancer.

### IARC disclaimer

Where authors are identified as personnel of the International Agency for Research on Cancer/World Health Organization, the authors alone are responsible for the views expressed in this article and they do not necessarily represent the decisions, policy or views of the International Agency for Research on Cancer/World Health Organization.

## Supporting information

S1 Table Baseline characteristics of men and women according to BMI categories in the UK Biobank cohort (n = 214,293 men and 244,420 women). (DOCX)

S2 Table Baseline characteristics of women by hormonal replacement therapy in the UK Biobank cohort (n = 244,420). (DOCX)

## Abbreviations

BMI: body mass index
HR: hazard ratio
CI: confidence interval
