# Supplementary material for: Anthropometry, body fat composition and reproductive factors and risk of oesophageal and gastric cancer by subtype and subsite in the UK Biobank cohort
Source: PLoS One. 2020 Oct 20;15(10):e0240413. doi: 10.1371/journal.pone.0240413 (PMC7575071; doi:10.1371/journal.pone.0240413)
Supplement: S1 Table — (DOCX) [file pone.0240413.s001.docx]

S1 Table. Baseline characteristics of men and women according to BMI categories in the UK Biobank cohort (n=214,293 men and 244,420 women)

|  |  |  | **BMI (kg/m^2^)^a^** | | | | | |  | | |
| --- | --- | --- | --- | --- | --- | --- | --- | --- | --- | --- | --- |
|  |  | Men |  |  |  |  | | Women | |  |  |
|  | Underweight  (BMI <18.5) | Normal weight  (18.5≤BMI<25) | Overweight (25≤BMI<30) | Obese  (BMI ≥30) |  | Underweight  (BMI <18.5) | | Normal weight  (18.5≤BMI<25) | | Overweight (25≤BMI<30) | Obese  (BMI ≥30) |
|  | (n=499) | (n=52,914) | (n=104,884) | (n=54,122) |  | (n=1828) | | (n=94,448) | | (n=88,945) | (n=57,526) |
| **Age at recruitment^b^ (years)** | 56 (49-61) | 57 (49-63) | 58 (50-63) | 58 (50-63) |  | 55 (48-62) | | 56 (48-62) | | 58 (51-63) | 58 (51-63) |
| **Height^b^ (cm)** | 175 (170-181) | 176 (171.4-181) | 176 (171-180) | 175 (171-180) |  | 164 (160-168) | | 163 (159-167.5) | | 162 (158-166) | 161 (157-165.5) |
| **Waist circumference^b^ (cm)** | 73 (69-77) | 86 (82-90) | 96 (92-100) | 109 (104-115) |  | 64 (62-67) | | 74 (70-79) | | 85 (81-90) | 99 (93-106) |
| **Hip circumference^b^ (cm)** | 88 (85-91) | 97 (94-100) | 103 (100-106) | 110 (107-115) |  | 86 (84-89) | | 96 (92-99) | | 103 (100-107) | 115 (110-121) |
| **Total body fat (%)** | 11.7 (9.4-14.5) | 19.7 (16.7-22.6) | 25.2 (22.7-27.7) | 31 (28.5-33.7) |  | 20.3 (17.3-23.2) | | 31.2 (27.8-34.1) | | 38 (35.6-40.3) | 44.4 (42-44.4) |
| **Trunk fat (%)** | 12.2 (8.2-16.5) | 21.7 (17.7-25.2) | 27.8 (24.8-30.6) | 34.1 (31.3-36.9) |  | 16.4 (12.5-20.1) | | 28.5 (24.3-32.2) | | 35.9 (32.6-39.0) | 42.1 (38.9-45.3) |
| **Education (%)** |  |  |  |  |  |  | |  | |  |  |
| None | 23.1 | 12.9 | 16.2 | 21.5 |  | 11.7 | | 11.9 | | 18.0 | 21.5 |
| CSEs/O-levels/GCSEs or equivalent | 18.2 | 21.1 | 24.3 | 26.3 |  | 22.5 | | 26.9 | | 29.2 | 29.9 |
| Vocational qualifications | 21.6 | 17.9 | 19.4 | 20.1 |  | 15.0 | | 16.3 | | 16.3 | 16.3 |
| Other qualifications | 2.4 | 3.8 | 4.4 | 4.9 |  | 4.7 | | 5.2 | | 5.9 | 6.1 |
| College/university | 32.1 | 42.5 | 33.7 | 25.1 |  | 43.4 | | 38.2 | | 28.7 | 23.9 |
| Missing/unknown | 2.6 | 1.9 | 1.9 | 2.2 |  | 2.7 | | 1.6 | | 1.9 | 2.3 |
| **Smoking (%)** |  |  |  |  |  |  | |  | |  |  |
| Never | 40.3 | 54.9 | 49.1 | 43.2 |  | 61.9 | | 61.7 | | 59.0 | 58.6 |
| Former | 17.4 | 29.5 | 38.6 | 44.8 |  | 20.7 | | 28.8 | | 31.9 | 32.6 |
| Current | 41.9 | 15.2 | 11.8 | 11.4 |  | 16.9 | | 9.1 | | 8.6 | 8.2 |
| Missing/unknown | 0.4 | 0.4 | 0.5 | 0.7 |  | 0.5 | | 0.4 | | 0.5 | 0.6 |
| **Alcohol intake frequency (%)** |  |  |  |  |  |  | |  | |  |  |
| Never | 13.6 | 6.7 | 5.7 | 7.1 |  | 16.0 | | 7.6 | | 8.9 | 13.2 |
| Special occasions only | 9.6 | 7.2 | 6.4 | 9.1 |  | 15.9 | | 11.4 | | 14.5 | 21.6 |
| 1-3 times/month | 9.4 | 8.3 | 8.3 | 10.7 |  | 10.0 | | 11.4 | | 12.9 | 15.9 |
| 1-2 times/week | 21.8 | 24.2 | 25.7 | 28.0 |  | 18.0 | | 25.8 | | 26.7 | 24.4 |
| 3-4 times/week | 18.0 | 25.9 | 27.5 | 23.7 |  | 18.7 | 23.9 | | 20.9 | | 14.7 |
| Daily or mostly | 26.1 | 27.4 | 26.2 | 21.2 |  | 21.0 | 19.8 | | 15.8 | | 9.8 |
| Missing/unknown | 1.4 | 0.3 | 0.2 | 0.3 |  | 0.4 | 0.2 | | 0.2 | | 0.3 |

^a^ BMI was classified according to World Health Organization categories: underweight (BMI <18.5 kg/m2), normal weight (18.5 ≤ BMI <25 kg/m2), overweight (25 ≤ BMI <30 kg/m2) and obese (≥30 kg/m2); 1,874 men and 1,673 women were missing BMI

^b^ Values are median (IQR, Q1-Q3); Differences between BMI categories were significant for all variables (<.0001)

Abbreviation: CSEs/O levels/GCSEs, Certificate of Secondary Education/General Certificate of Secondary Education or equivalent
