# Supplementary material for: Anthropometry, body fat composition and reproductive factors and risk of oesophageal and gastric cancer by subtype and subsite in the UK Biobank cohort
Source: PLoS One. 2020 Oct 20;15(10):e0240413. doi: 10.1371/journal.pone.0240413 (PMC7575071; doi:10.1371/journal.pone.0240413)
Supplement: S2 Table — (DOCX) [file pone.0240413.s002.docx]

S2 Table. Baseline characteristics of women by hormonal replacement therapy

in the UK Biobank cohort (n=244,420)

|  | | Hormonal replacement therapy | | | | | | | |  |
| --- | --- | --- | --- | --- | --- | --- | --- | --- | --- | --- |
|  | | Yes | | | | | No | | |  |
|  | |  | | | | |  | | |  |
| **Age at recruitment^a^ (years)** |  | | 61 (65-67) | | | | | 53 (47-60) | |  |
| **BMI^a^ (kg/m^2^)** |  | | 26.5 (23.9-29.9) | | | | | 25.9 (23.2-29.6) | |  |
| **Waist circumference^a^ (cm)** |  | | 84 (77-93) | | | | | 82 (75-92) | |  |
| **Hip circumference^a^ (cm)** |  | | 101 (96-108) | | | | | 102 (97-109) | |  |
| **Total body fat (%)** |  | | 37.6 (33.2-41.9) | | | | | 36.2 (31.3-41.0) | |  |
| **Trunk fat (%)** |  | | 35.3 (30.3-40.1) | | | | | 33.9 (28.3-39.2) | |  |
| **Education (%)** |  | |  | | | | | |  | |
| None |  | | 22.8 | | | | | | 12.6 |  |
| CSEs/O-levels/GCSEs or equivalent |  | | 27.7 | | | | | | 28.9 |  |
| Vocational qualifications | | | | |  | 15.2 | | | 16.9 |  |
| Other qualifications | |  | | 7.1 | | | | | 4.8 |  |
| College/university | |  | | 25.3 | | | | | 35.1 |  |
| Missing/unknown | |  | | 1.9 | | | | | 1.6 |  |
| **Smoking status (%)** | |  | |  | | | | |  |  |
| Never | |  | | 54.0 | | | | | 63.6 |  |
| Former | |  | | 36.6 | | | | | 27.4 |  |
| Current | |  | | 9.0 | | | | | 8.7 |  |
| Missing/unknown | |  | | 0.4 | | | | | 0.3 |  |
| **Alcohol intake frequency (%)** | |  | |  | | | | |  |  |
| Never | |  | | 9.2 | | | | | 9.6 |  |
| Special occasions only | |  | | 15.4 | | | | | 14.8 |  |
| 1-3 times/month | |  | | 12.5 | | | | | 13.5 |  |
| 1-2 times/week | |  | | 24.7 | | | | | 26.4 |  |
| 3-4 times/week | |  | | 19.9 | | | | | 21.0 |  |
| Daily or mostly | |  | | 18.2 | | | | | 14.6 |  |
| Missing/unknown | |  | | 0.07 | | | | | 0.08 |  |

**^a^** Values are median (IQR, Q1-Q3)

Abbreviation: CSEs/O levels/GCSEs, Certificate of Secondary Education/General Certificate of Secondary Education or equivalent
